# Targeting lysosomal ion channels to overcome cancer treatment resistance: emerging mechanisms and therapeutic opportunities

**DOI:** 10.3389/fphar.2026.1911117

**Published:** 2026-09-25

**Authors:** Na Luo, Qiuxiang Wang, Jun Zhang, Zhiqin Deng

**Affiliations:** 1 Department of Critical Care Medicine, The First Affiliated Hospital of Chongqing Medical University, Chongqing, China; 2 Chongqing Key Laboratory of Intelligent Oncology for Breast Cancer, Center for Intelligent Oncology, Chongqing University Cancer Hospital, School of Medicine, Chongqing University, Chongqing, China; 3 Clinical Center for Tumor Therapy, The Second Affiliated Hospital, Chongqing Medical University, Chongqing, China

**Keywords:** drug resistance, immunotherapy resistance, ion channel, lysosome, radiotherapy resistance, tumor therapy

## Abstract

Lysosomes are critical cellular organelles that not only regulate metabolic regulation but also play pivotal roles in cell death, signal transduction, and tumorigenesis. The various ion channels expressed on the lysosomal membrane are central to these physiological functions; they intricately maintain lysosomal pH homeostasis, calcium ion homeostasis, and membrane potential. Consequently, the dysregulation of these channels has emerged as a potential target of tumor cells, driving tumor cell proliferation, metastasis, apoptosis, and drug resistance. This review summarizes the structure and function of major lysosomal ion channels, emphasizing their central roles in regulating the tumor microenvironment, cell survival, and treatment resistance. Specifically, we highlight their involvement in mediating resistance to chemotherapy, radiotherapy, and immunotherapy, elucidating the associated molecular mechanisms. Furthermore, we explore the prospects and challenges of developing targeted therapeutic strategies towards these ion channels to overcome tumor drug resistance.

## 1 Introduction

### 1.1 Overview of lysosomes

First identified by Christian de Duve in 1950 within mouse liver cells, the lysosome is a membrane-bound organelle characterized by an abundance of hydrolytic enzymes (De duve et al., 1955; Mindell, 2012; Coffey and De duve, 1968). Serving as the primary hub for intracellular material and energy recycling, lysosome contains more than 60 types of hydrolytic enzymes that facilitate the degradation of macromolecules, thus playing a significant role in cellular metabolism. Specifically, lysosomes process extracellular nutrients internalized via endocytosis (Okamoto, 2014; Luzio et al., 2009), as well as intracellular macromolecules and organelles delivered through autophagy (Kaushik and Cuervo, 2012). Beyond these canonical degradative functions, recent studies have revealed that lysosomes also execute other critical functions, including sensing cellular energy status, facilitating plasma membrane repair, regulating cellular signaling pathways, maintaining cellular protein homeostasis, and modulating immune response (Settembre et al., 2013; Ballab et al., 2020; Li et al., 2016). Consequently, lysosomes have transcended their traditional function as mere terminal degradation centers, emerging as critical signaling centers for cellular metabolic sensing and signal transduction.

### 1.2 Overview of lysosomal ion channels

To execute these complex degradative and signaling functions, the lysosome relies on the dynamic ion balance mediated by membrane ion channels. The advent and refinement of lysosomal patch-clamp electrophysiology techniques have enabled the precise characterization of these channels. By regulating the transmembrane flux of ions (e.g., H^+^, Ca^2+^, K^+^, Cl^−^), they are indispensable for maintaining ion gradients, lysosomal pH homeostasis, membrane potential, and modulating intracellular signaling pathways (Figure 1) (Kondratskyi et al., 2025). Consequently, genetic mutations for protein dysfunctions of lysosomal ion channels are closely associated with a variety of human diseases (Jeong et al., 2022; Wang et al., 2019; Zhu et al., 2026). In recent years, as these ion channels have been increasingly characterized, their massive involvement in tumor biology has become highly apparent, suggesting them as potential targets for tumor treatment.

**FIGURE 1:**
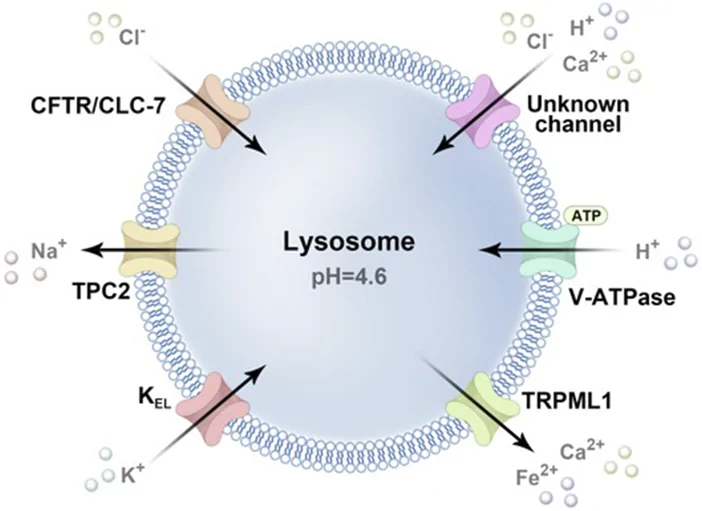
Types of ion channels on the lysosomal membrane.

## 2 Lysosomal ion channels: structure and function in cancer cells

### 2.1 Calcium ion channels

Calcium is a ubiquitous and diffusible second messenger that plays a critical role in cellular signaling pathways, coordinating many fundamental physiological processes in tumor progression. Within the lysosomal compartment, the transient receptor potential mucolipin (TRPML) family, comprising TRPML1, TRPML2, and TRPML3, serves as the primary Ca^2+^-release channels. As nonselective cation channels, they are primarily responsible for releasing Ca^2+^ from the lysosomal lumen into the cytoplasm (Li et al., 2019; Schmiege et al., 2017; Fine et al., 2018). This process is essential for maintaining the lysosomal ion homeostasis, facilitating autophagosomes-lysosome fusion, regulating lysosomal motility, and initiating lysosomal biogenesis. Given these important functions, dysregulation of TRPML channels is closely linked to tumor cell survival and treatment resistance. For instance, studies have revealed that inhibiting TRPML1 can induce ferroptosis in breast cancer stem cells (CSCs). By eliminating these therapy-resistant CSCs, which are highly prone to driving tumor recurrence, TRPML1 inhibitors can significantly suppress tumor growth and enhance the sensitivity of cancer cells to chemotherapeutic agents (Fan et al., 2024; Grimm et al., 2018). Similarly, melanoma cells lacking TRPML1 exhibit reduced survival, proliferation, tumor growth, and macropinocytosis, accompanied by serine depletion and proteotoxic stress. Moreover, TRPML1 negatively regulates extracellular-signal-regulated kinase (ERK) phosphorylation and mTORC1 activity in melanoma (Kasit et al., 2019). At the molecular level, TRPML1-dependent lysosomal Ca^2+^ release activates downstream calcineurin signaling, triggering transcription factor EB (TFEB) dephosphorylation and nuclear translocation to initiate transcription of autophagy-lysosome-related genes. The TRPML1-calcineurin-TFEB axis mediates chemoresistance in tumor cells by sustaining lysosomal biogenesis and enhancing lysosomal drug sequestration; inhibition of TRPML1 or calcineurin blocks TFEB activation and nuclear import to reverse drug resistance and potentiate chemotherapy (Medina et al., 2015). Accordingly, TFEB/TFE3 serve as central transcriptional hubs linking TRPML1-derived calcium signals to adaptive survival programs underlying tumor drug resistance.

P2X4, a key P2X-family lysosomal ATP-gated Ca^2+^ channel, functions as a trimeric ionotropic receptor. Highly sensitive to ATP, it preferentially resides in lysosomal and endolysosomal compartments, where heavy glycosylation maintains its stability in the lysosomal microenvironment (Suurväli et al., 2017). Activated by luminal ATP (ATP^4-^) under near-neutral pH, P2X4 triggers lysosomal Ca^2+^ efflux into the cytosol. Via calmodulin signaling, it facilitates lysosomal membrane fusion and acts as a central hub governing lysosomal trafficking, fusion and intracellular positioning (Suurväli et al., 2017; Tan et al., 2021). Preclinical evidence highlights its implications in various malignancies, including promoting prostate cancer progression (He JP et al., 2020), facilitating colorectal cancer metastasis (Zhou et al., 2025), and driving hepatocellular carcinoma progression via the Ca^2+^-PI3K/AKT signaling pathway (Wang et al., 2025). Consequently, targeting both TRPML1 and P2X4 emerges as a promising strategy to eliminate cancer stem cells and enhance chemosensitivity (Schmitt et al., 2022; Lyu et al., 2020; Jung and Venkatachalam, 2019).

### 2.2 Sodium ion channels

Within the tumor microenvironment, the concentration of sodium ions is significantly higher than in normal tissues. This gradient is closely associated with the formation and maintenance of the tumor immune microenvironment and interacts with the complex biological processes underlying tumorigenesis (Cardone et al., 2005). As key sodium-permeable channels localized to the endolysosomal system, two-pore channels (TPCs), as key sodium ion channels located on the lysosomal system, play a complex and crucial role in tumor development and progression. They not only serve as hubs for intracellular signal transduction but also act as critical molecular regulators of tumor proliferation, immune evasion, and metabolic reprogramming (Wang et al., 2012; Gan and Jiang, 2022). For example, studies have revealed that inhibiting TPC2 exerts potent anti-tumor effects in hepatocellular carcinoma. On one hand, TPC2 promotes the proliferation of liver cancer cells by regulating tumor energy metabolism (Müller et al., 2021). On the other hand, TPC2 inhibition significantly enhances the cytotoxicity of CD8^+^ T cells, effectively activating the host immune system to bolster anti-tumor immunity, thereby enabling more efficient tumor recognition and elimination (Ouologuem et al., 2025). Additionally, the sodium channel subunit SCNN1B has also been identified as a potential anticancer target, its activation can trigger the unfolded protein response by degrading GRP78, which in turn activates PERK, ATF4, XBP1s, and C/EBP homologous proteins, ultimately leading to cancer cell apoptosis (Qian et al., 2017).

### 2.3 Potassium ion channels

Potassium channels represent the most diverse and extensively studied family of ion channels, playing a critical role in cell proliferation, apoptosis, cell volume regulation, and maintenance of membrane potential. Potassium channels are primarily classified into four major types: voltage-gated potassium channels, calcium-activated potassium channels, inwardly rectifying potassium channels, and two-pore domain potassium channels (Pardo and Stühmer, 2014). Among them, the large-conductance calcium-activated potassium (BK) is activated by both membrane potential and intracellular Ca^2+^ concentration (Wang et al., 2017; Cao et al., 2015). In the context of glioblastoma (GBM), a glioma-specific BKCa splice variant (gBKCa) is abnormally highly expressed (Maliszewska-olejniczak et al., 2025). During invasion, tumor cells actively efflux K^+^ via gBKCa channels, working in concert with chloride channels to facilitate water removal, which reduce cell volume and enable them to penetrate the extracellular matrix, thereby promoting tumor metastasis. Furthermore, under hypoxic conditions and in the presence of chemotherapeutic drug toxicity, gBKCa maintains tumor cell survival by modulating mitochondrial membrane potential (Maliszewska-olejniczak et al., 2025; Zhang et al., 2025a). Collectively, potassium channels not only serve as crucial sensors for tumor cells to perceive microenvironment change and maintain their survival but also act as key molecular drivers underlying tumor invasion, metabolic reprogramming, and therapeutic resistance (Bortner and Cidlowski, 2014; Li et al., 2022).

### 2.4 Chloride ion channels

Lysosomal chloride channels are essential components for maintaining lysosomal homeostasis. The key membrane proteins known to mediate chloride ion permeability across the lysosomal membrane primarily include the cystic fibrosis transmembrane conductance regulator (CFTR) and chloride channel protein 7 (CLC-7) (Bradbury, 1999; Kasper et al., 2005; Wang et al., 2021; Sharaireh et al., 2025). These two proteins finely regulate the lysosomal ion environment, membrane potential, and acid-base balance through distinct molecular mechanisms, thereby influencing lysosomal maturation, fusion, and enzymatic activity. Consequently, they play crucial roles in tumor proliferation, invasion, metastasis, and immune evasion. Beyond its canonical role as a chloride channel maintaining lysosomal function, CFTR also participates in the regulation of intracellular immune signaling pathways. For instance, in lung cancer and colorectal cancer, the loss of CFTR leads to impaired lysosomal acidification and blockage of autophagic flux, which in turn promotes the survival and proliferation of tumor cells. Moreover, the functional status of CFTR is associated with immune cell infiltration in the tumor microenvironment, it may modulate cytokine secretion and thus contribute to antitumor immune surveillance (Duan et al., 2021; Caverly et al., 2022). Therefore, lysosomal chloride channels maintain lysosomal functional homeostasis via diverse mechanisms, and abnormalities in their expression or function are closely linked to the initiation and progression of various tumors. Although currently approved CFTR-targeted agents are indicated for non-oncological diseases, the clinical maturity of CFTR modulators (such as ivacaftor and the elexacaftor class) in treating cystic fibrosis (Lopes-pacheco, 2020), demonstrates the pharmacological viability of channel-directed drug design and lays a theoretical foundation for drug repurposing and optimization of lysosomal channel directed antitumor therapies. Recent studies reveal that the CFTR potentiator ivacaftor synergistically reverses acquired osimertinib resistance in EGFR-mutant non-small-cell lung cancer via the CFTR-PTEN-AKT signaling axis (Li YK et al., 2025). Repurposing these mature modulators or utilizing them as structural scaffolds offers a opportunities for tumor treatment by restoring lysosomal autophagic flux and reinvigorating antitumor immune surveillance.

## 3 The role of lysosomal ion channels in tumor treatment resistance

Chemotherapy, radiotherapy, and immunotherapy have become the main approaches for malignant tumor treatment. However, the therapeutic outcomes are limited by resistance developed by tumor cells. Lysosomal ion channels play a pivotal role in mediating this resistance by regulating intraluminal pH, maintaining membrane potential, and modulating autophagic flux, which collectively facilitate intracellular drug sequestration, apoptosis escape, and immune evasion. Consequently, targeting lysosomal ion channels has emerged as a highly promising therapeutic strategy to overcome treatment resistance. The combination of ion channel modulators with conventional anticancer drugs can produce synergistic effects, enhancing treatment sensitivity and overcoming drug resistance (Shi et al., 2025).

### 3.1 Research progress on lysosomal ion channels in chemotherapy resistance

Multidrug resistance (MDR) represents a formidable challenge in cancer chemotherapy. Its mechanisms involve the hyperactivation of drug efflux pumps, suppression of apoptotic pathways, enhanced DNA damage repair, and dynamic remodeling of the tumor microenvironment. In recent years, with deepening insights into the electrophysiological properties of tumor cells, the critical role of the aberrant expression and dysfunction of lysosomal ion channels in the development of MDR has gradually come to light. These channels not only maintain fundamental electrochemical homeostasis but also act as pivotal signaling nodes. By modulating the local ionic microenvironment, they are deeply involved in regulating cell proliferation, migration, death, and drug distribution, serving as an “invisible switch” that dictates tumor cell fate and drug sensitivity (Zong et al., 2016; Kondratskyi et al., 2025; Zhitomirsky et al., 2016; Fan et al., 2024).

#### 3.1.1 Lysosomal ion channels and drug sequestration mechanisms

As the cell’s intracellular degradation center, the lysosome’s function is highly dependent on the acidic pH and membrane potential maintained by ion channels in its membrane. In drug-resistant tumor cells, lysosomes often exhibit a phenomenon known as “drug trapping,” in which weakly basic chemotherapeutic agents are sequestered within the lysosomal lumen via enhanced endocytosis or membrane transport mechanisms, preventing them from reaching their cellular targets such as the nucleus and exerting their therapeutic effects (Zhitomirsky and Assaraf, 2016). Ion channels play a crucial role in this process. For example, the loss of function of CFTR or the altered expression of CLC-7 leads to alterations in lysosomal chloride ion permeability, which in turn causes intraluminal pH imbalance and disruption of membrane potential (Luciani et al., 2010; Graves et al., 2008). Such disruption of ionic homeostasis not only impairs the activity of lysosomal hydrolases but may also inhibit the normal flow of the autophagic-lysosomal pathway, resulting in the accumulation of damaged organelles and misfolded proteins and helping tumor cells evade drug-induced programmed cell death. Studies have shown that loss of CFTR function or abnormal expression of CLC-7 can lead to lysosomal pH imbalance, affecting drug release and degradation and thereby promoting drug resistance (Lee et al., 2020; Zhitomirsky et al., 2016). Pharmacological inhibition or genetic silencing of these channels can often restore the sensitivity of cancer cells to chemotherapeutic drugs, offering a promising new approach for reversing multidrug resistance. For example, preclinical studies have demonstrated that the application of the TRPML1-specific inhibitor ML-SI1 significantly sensitizes drug-resistant breast cancer stem cells to doxorubicin. By blocking TRPML1, ML-SI1 disrupts lysosomal function, leading to the accumulation of reactive oxygen species and triggering ferroptosis, thereby overcoming chemoresistance (Fan et al., 2024). Furthermore, tetrandrine, a naturally occurring alkaloid and a potent inhibitor of TPCs, has been successfully utilized in preclinical models of multidrug-resistant cancer. Studies show that tetrandrine effectively reverses doxorubicin resistance by preventing the lysosomal sequestration of these weakly basic chemotherapeutics, allowing them to reach their nuclear targets and exert their cytotoxic effects (Xin et al., 2025). Beyond TRPML1, other members of the TRPML family also play critical roles in tumor progression and therapeutic resistance by governing lysosomal calcium efflux. Accumulating evidence demonstrates that TRPML3-dependent lysosomal Ca^2+^ release drives osimertinib (EGFR-TKI) resistance in non-small-cell lung cancer. By enhancing the lysosomal sequestration of osimertinib, TRPML3 prevents the drug from reaching its intracellular targets, serving as a textbook example of how specific lysosomal channels mediate targeted therapy resistance via drug trapping (Kim and Kim, 2025). Therefore, targeting lysosomal ion channels has emerged as a novel strategy for overcoming chemotherapy resistance and enhancing therapeutic efficacy.

#### 3.1.2 Mitochondrial ion homeostasis and the mechanism of apoptosis escape

Mitochondria serve as a central hub for regulating apoptosis, and their membrane potential and ionic homeostasis directly determine the cell’s fate—whether it lives or dies. In the context of drug resistance, many tumor cells exhibit electrophysiological characteristics such as mitochondrial membrane hyperpolarization and downregulation of potassium channel expression. This “high membrane potential, low permeability” state makes mitochondrial outer membrane permeabilization (MOMP) highly unlikely, thereby blocking the release of cytochrome c and the initiation of the downstream apoptotic cascade. Studies have shown that restoring potassium ion influx into mitochondria can effectively neutralize the membrane potential, induce opening of the mitochondrial permeability transition pore (mPTP), and trigger a burst of reactive oxygen species (ROS), inhibit ATP synthesis, and activate pro-apoptotic proteins such as Bax and Bak (Singh et al., 2019; Ma et al., 2024). This strategy—artificially intervening in ion flux to “restart” the mitochondrial apoptosis machinery—offers a novel approach to overcoming drug resistance.

### 3.2 Lysosomal ion channels and radiotherapy resistance

Radiotherapy (RT), one of the cornerstone modalities in cancer treatment, primarily exerts its effect by directly inducing double-strand DNA breaks in tumor cells via high-energy radiation or, indirectly, by generating large amounts of reactive oxygen species (ROS) through water radiolysis, thereby triggering cell death. However, intrinsic or acquired radioresistance of tumor cells significantly limits the clinical efficacy of radiotherapy. Recent studies have revealed that ion channels on the lysosomal membrane play a critical role in sensing radiation signals, maintaining cellular homeostasis, and mediating resistance. Research indicates that specific lysosomal ion channels can influence the radiosensitivity of tumor cells by regulating autophagy and apoptosis (Figure 2) (Lu et al., 2025; Kendall and Holian, 2021).

**FIGURE 2:**
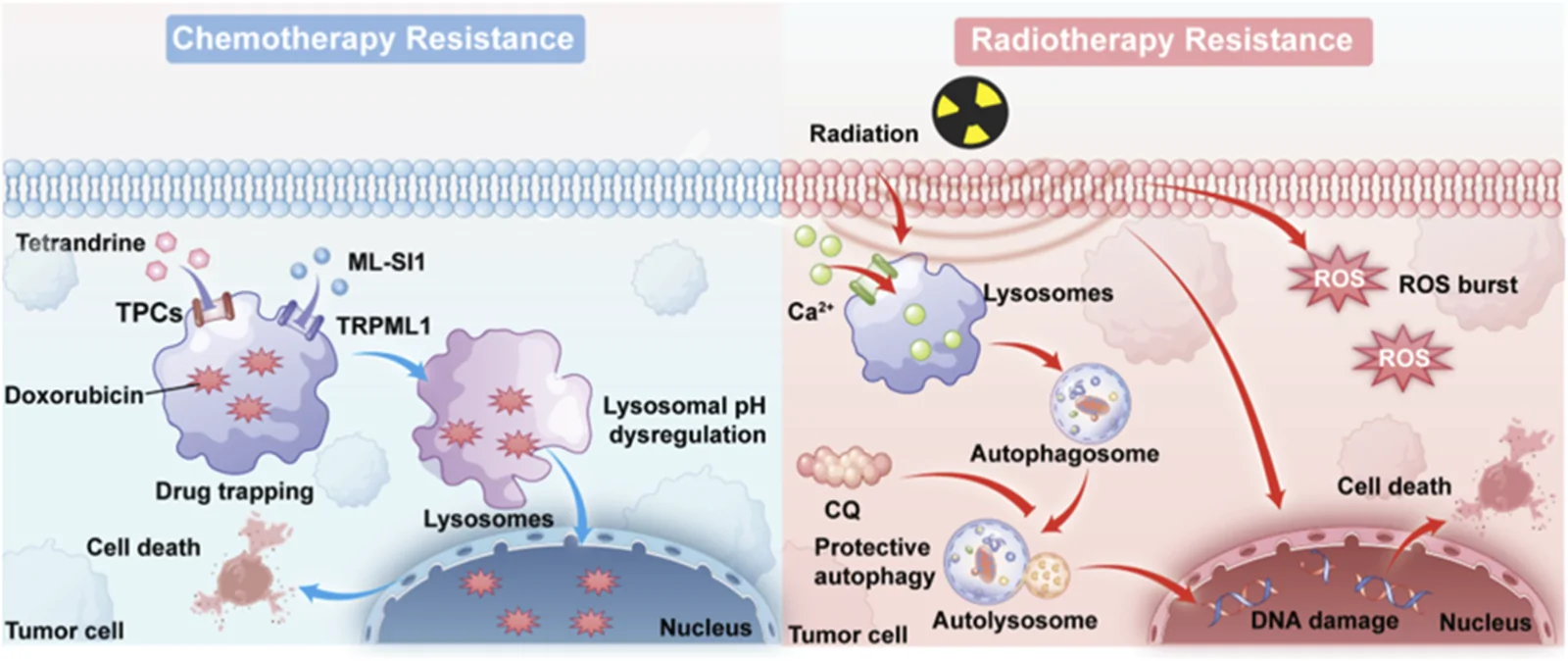
Mechanism of lysosomal ion channels in mediating chemotherapy and radiotherapy resistance.

#### 3.2.1 Lysosomal calcium channels and radiotherapy resistance

Studies have found that abnormal activation of lysosomal calcium channels can lead to an increase in intracellular calcium concentration, which in turn activates the autophagy signaling pathway, enabling tumor cells to survive after radiotherapy and exhibit enhanced resistance. In certain tumor models, excessive activation of lysosomal calcium channels can promote protective autophagy, allowing tumor cells to clear damaged organelles and protein aggregates, thereby enhancing their survival capacity and reducing the effectiveness of radiotherapy. In glioblastoma, inhibiting the function of lysosomal calcium channels can effectively block the autophagy flux, thus enhancing the tumor cells’ response to radiation. The transient receptor potential vanilloid subtype 1 (TRPV1) is a calcium channel that is overexpressed in various types of tumor cells. Activation of the TRPV1 channel can facilitate the influx of extracellular calcium ions, leading to intracellular calcium dysregulation. Studies have shown that activating the TRPV1 channel to induce calcium overload can disrupt the homeostasis of tumor cells, inhibit DNA damage repair, and enhance radiosensitivity (Wang et al., 2024; D'alessandro et al., 2018).

#### 3.2.2 Lysosomal hydrogen ion channels and radiotherapy resistance

Lysosomal proton channels play a pivotal role in maintaining lysosomal function and regulating radioresistance in tumor cells. The extremely low intra-lysosomal pH is a critical factor for their degradative capacity, and the establishment of this acidic environment relies primarily on the combined action of proton pumps and proton channels on the lysosomal membrane. Studies have shown that dysfunction of proton channels can lead to an increase in lysosomal pH, thereby impairing the activity of hydrolytic enzymes within the lysosome and consequently weakening its ability to degrade extracellular particles and intracellular components. In tumor cells, this decline in degradation capacity may further affect drug uptake and DNA damage repair processes, ultimately contributing to the development of radioresistance. Moreover, proton channels also influence the sensitivity of tumor cells to radiation therapy by regulating the dynamic interactions between lysosomes and the cell membrane. For instance, in breast cancer cells, activation of proton channels can promote the fusion of lysosomes with the cell membrane, enhancing the tumor cells’ ability to take up exogenous drugs and increasing their sensitivity to radiation (Wu et al., 2023; Wu et al., 2020). In a specific therapeutic case, the use of lysosomotropic agents such as chloroquine (CQ), which accumulates in lysosomes and neutralizes their acidic pH, has been successfully combined with radiotherapy. Preclinical studies in glioblastoma models revealed that the addition of CQ to standard radiation therapy significantly prolonged tumor growth delay. This synergistic effect was achieved by blocking radiation-induced protective autophagy, thereby exacerbating DNA damage and promoting cancer cell apoptosis (Wang et al., 2018).

#### 3.2.3 Lysosomal potassium channels and radiotherapy resistance

Recent studies have shown that transmembrane protein 175 (TMEM175), an important lysosomal potassium ion channel, plays a role in regulating the ionic homeostasis and membrane potential of lysosomes, thereby influencing lysosomal function and its interactions with cellular signaling networks. In tumor cells, the expression level of TMEM175 often undergoes significant changes, which may be closely associated with their radioresistance phenotype. Expression profiling studies have confirmed that TMEM175 is highly expressed in glioblastoma tissues (Puchalski et al., 2018). Notably, under conditions of irradiation stress, the functionality of TMEM175 becomes critical for cellular adaptation (Su et al., 2024). Therefore, in glioblastoma, the high expression of TMEM175 may be associated with reduced sensitivity to radiation therapy, as it may enhance lysosomal function or modulate cellular signaling pathways to promote survival under genotoxic stress.

#### 3.2.4 Lysosomal chloride channels and radiotherapy resistance

Chloride channels have also garnered significant attention due to their role in regulating lysosomal volume and osmotic pressure. Studies indicate that the activation of chloride channels facilitates chloride efflux from lysosomes, thereby influencing the processes of lysosomal swelling and rupture. In tumor cells, this heightened lysosomal membrane instability can trigger the release of cytotoxic substances, subsequently activating cell death pathways. By manipulating these mechanisms, targeting chloride channels could potentially sensitize tumors to radiation. Collectively, lysosomal ion channels modulate the response of tumor cells to radiotherapy by regulating intracellular ion homeostasis, cellular signaling, and cell death pathways (Zhang et al., 2018; Klumpp et al., 2018).

### 3.3 Lysosomal ion channels and immune therapy resistance

Immune checkpoint inhibitors (ICIs) represent an important therapeutic approach in cancer treatment; however, primary or acquired resistance remains widespread. Resistance to tumor immunotherapy involves both intrinsic mechanisms within tumor cells—such as defects in antigen presentation—and extrinsic mechanisms, including an immunosuppressive microenvironment. Tumor immunotherapy resistance is a complex, multifactorial process in which the abnormal expression and altered function of lysosomal ion channels play a crucial role. Acting as “signal commanders” in the tumor microenvironment, these ion channels profoundly influence tumor cell immune evasion capabilities and the antitumor efficacy of immune cells by regulating ion fluxes (Figure 3).

**FIGURE 3:**
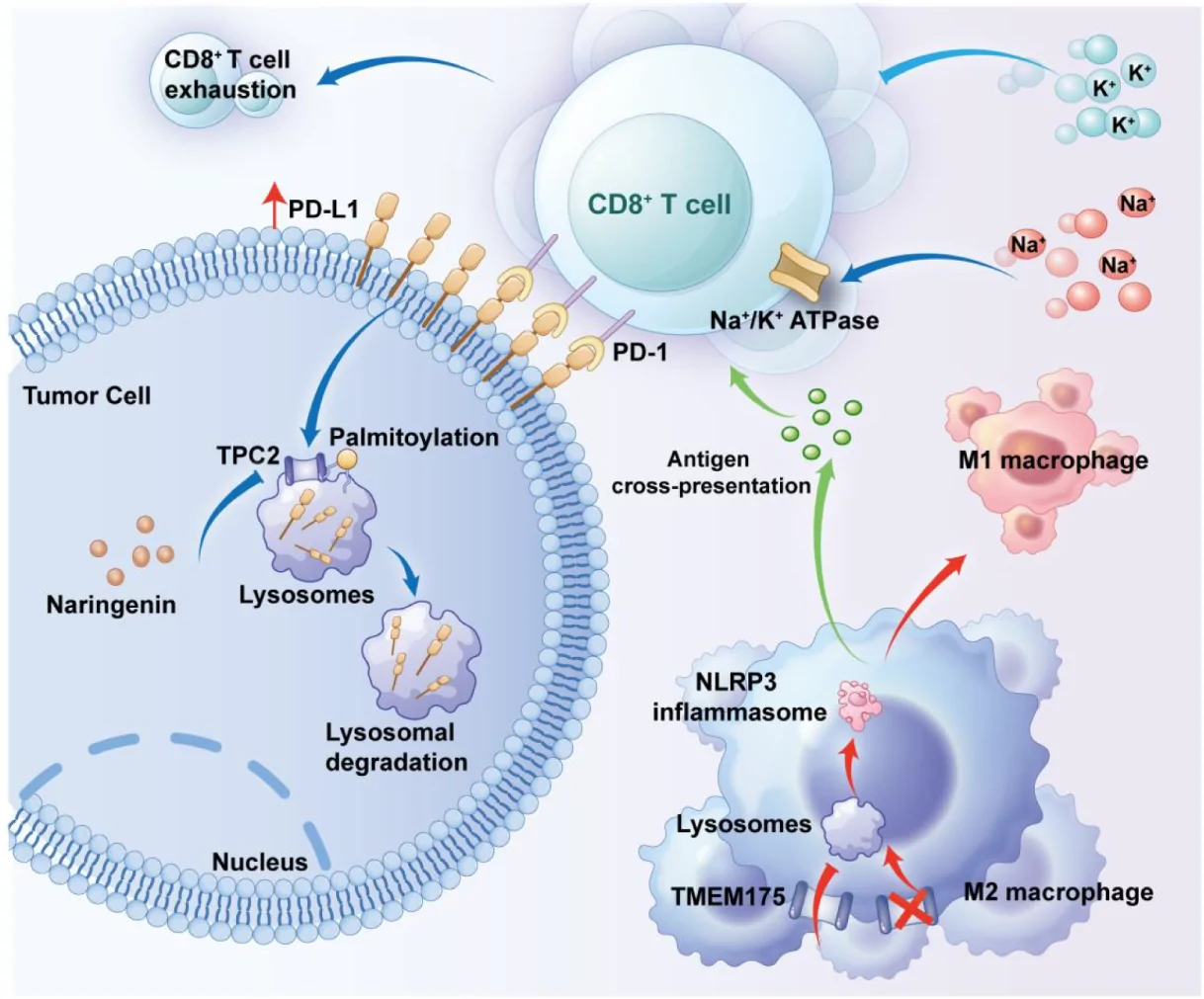
Crosstalk between lysosomal ion channels and the tumor immune microenvironment.

#### 3.3.1 Potassium ion channels

Potassium channels are central to maintaining membrane potential and cell volume, with their aberrant activity directly linked to immunotherapy resistance. In tumors like glioblastoma, the overexpression of specific voltage-gated potassium channels (e.g., BK channels) helps cancer cells maintain metabolic homeostasis and survive under stress (e.g., hypoxia). Furthermore, by modulating cell volume, these channels enhance the physical invasiveness required for tissue penetration and metastasis (Zhang et al., 2025b). In the tumor microenvironment, high extracellular potassium concentrations suppress cytotoxic T cells. Tumor cells release substantial K^+^ via these channels, elevating intracellular K^+^ in T cells. This inhibits their proliferation, cytokine production, and cytotoxicity, driving T cell “exhaustion” and rendering immune checkpoint inhibitors ineffective. Notably, the lysosomal potassium channel TMEM175 acts as a negative regulator in macrophage-mediated immunity. Macrophage-specific deletion of TMEM175 activates the NLRP3 inflammasome and enhances antigen cross-presentation. Mechanistically, the engulfment of tumor cell debris by Tmem175−/− macrophages causes lysosomal membrane permeabilization. This permeabilization, exacerbated by reduced lysosomal acidification and elevated cytosolic Ca^2+^, leads to the release of cathepsin B into the cytosol, which directly activates the NLRP3 inflammasome (Zhang et al., 2026). This remodels the immune microenvironment, promotes M1 macrophage polarization, and boosts T and NK cell functions, ultimately inhibiting tumor growth and significantly improving the response to anti-PD-1 therapy.

#### 3.3.2 Calcium ion channels

Calcium ions (Ca^2+^) serve as crucial intracellular second messengers, and the activity of the lysosomal calcium channel TPC2 directly influences autophagic flux and antigen presentation in tumor cells. Aberrant activation of the TPC2 channel disrupts lysosomal acidification, preventing autophagosomes from properly fusing with lysosomes—a phenomenon known as “autophagic flux blockage.” In this state, tumor cells accumulate large numbers of undegraded autophagosomes, which not only represent a survival strategy for coping with nutrient deprivation but are also associated with resistance to targeted therapies. The activity of TPC2 is closely linked to the stability of immune checkpoint molecules such as PD-L1 and B7-H4. Through mechanisms like palmitoylation modification, TPC2 helps these immune checkpoint molecules evade lysosomal degradation, enabling their sustained high expression on the surface of tumor cells. This continuous suppression of T-cell activity ultimately leads to immune evasion and therapeutic resistance (Yan et al., 2025; Ouologuem et al., 2025; Abrahamian et al., 2024; Zaidi and Soboloff, 2025). As a concrete therapeutic example, recent investigations have utilized naringenin, a natural flavonoid that modulates TPC2 activity. In melanoma mouse models, pharmacological intervention targeting TPC2 disrupted the lysosomal evasion of PD-L1, leading to its degradation. This targeted approach not only reduced the surface expression of PD-L1 on tumor cells but also significantly enhanced the infiltration and cytotoxicity of CD8^+^ T cells, demonstrating a profound synergistic effect when combined with anti-PD-1 immunotherapy (Geisslinger et al., 2022; Wu et al., 2025). Furthermore, lysosomal calcium channels are integral to mitochondrial-lysosomal crosstalk, which profoundly shapes antitumor immune cell function. For example, recent studies demonstrate that TRPML1 sustains the mitochondrial fitness of NK cells via inter-organelle Ca^2+^ transfer. This lysosome to mitochondria calcium signaling is essential for maintaining NK cell cytotoxicity and preventing immune exhaustion within the immunosuppressive tumor microenvironment (Clement et al., 2023).

#### 3.3.3 Sodium ion channels

While the tumor microenvironment is often characterized by high extracellular sodium concentrations, the intracellular handling of this sodium gradient relies heavily on lysosomal sodium channels. TPCs, particularly TPC1 and TPC2, are the primary sodium-permeable channels in the endolysosomal system. In the context of immunotherapy resistance, TPC-mediated sodium signaling regulates endolysosomal trafficking and the degradation of immune checkpoint proteins (Li S. et al., 2025). The high extracellular sodium in tumors, often maintained by plasma membrane transporters, can influence intracellular sodium stores, thereby modulating lysosomal TPC activity. This altered lysosomal sodium flux impacts the metabolic adaptability of immune cells and the surface presentation of tumor antigens. By bridging the broader sodium-rich tumor microenvironment directly to lysosomal ion homeostasis, TPC-mediated sodium signaling plays a fundamental role in driving immune evasion and shaping the efficacy of immunotherapies (He T. et al., 2020).

## 4 Conclusion and outlook

Lysosomal ion channels serve as pivotal nodes in the regulation of tumor metabolic reprogramming, profoundly influence tumor initiation, progression, metastasis, and treatment resistance through the precise maintenance of lysosomal pH and ionic homeostasis. Mechanistically, these channels orchestrate a complex network of cellular processes by regulating the autophagy-lysosome pathway, driving metabolic adaptations, facilitating immune evasion via altered antigen presentation, and promoting physical invasion. Consequently, targeting these critical channels offers a compelling strategy to reshape both the tumor metabolome and the immune microenvironment, providing novel avenues to overcome therapeutic resistance.

Targeting lysosomal ion channels represents a promising frontier in anticancer drug development, with the core strategy revolving around exploiting the unique functional characteristics of lysosomes to disrupt ion homeostasis and induce tumor cell death. One primary approach involves utilizing small-molecule modulators to directly activate or inhibit endogenous ion channels on the lysosomal membrane, thereby altering lysosomal functional states to trigger targeted cell death. Another strategy leverages the highly acidic nature of tumor lysosomes to selectively accumulate drugs through the “lysosomotropic trap” mechanism or employs alkalinizing agents to neutralize lysosomal pH, both of which disrupt the metabolic balance and autophagic flux of tumor cells. Furthermore, an innovative direction entails engineering artificial ion channels via chemical synthesis or supramolecular self-assembly. These synthetic channels are designed to insert into the lysosomal membrane and activate specifically within the acidic tumor microenvironment, achieving selective cancer cell killing by catastrophically disrupting intracellular ion homeostasis. Preclinical studies have already demonstrated that targeting channels such as TRPML1, TPCs, and P2X4 via these strategies can successfully restore the sensitivity of refractory cancers to conventional chemo- and immunotherapies.

Despite these promising preclinical advancements, the clinical translation of lysosomal ion channel modulators faces several formidable challenges. A representative example is the clinical trajectory of lysosomotropic agents such as hydroxychloroquine (HCQ), which remains the only lysosome-targeting strategy with a substantial clinical trial history in oncology. Phase I trials combining HCQ with agents such as vorinostat, temsirolimus, temozolomide, and bortezomib established tolerability but showed limited efficacy signals (Poklepovic and Gewirtz, 2014). This pattern has largely persisted in more recent trials, such as HCQ combined with palbociclib and letrozole in ER+/HER2-breast cancer (NCT03774472). These clinical outcomes suggest that simple lysosomal alkalinization is insufficient and highlight the necessity for more specific channel modulators. Furthermore, a major hurdle is target selectivity, as many lysosomal channels, such as P2X4, share high homology with counterparts on the plasma membrane or other organelles, making it difficult to design drugs that exclusively target the lysosomal isoforms without triggering off-target effects. Additionally, achieving efficient, stable, and specific delivery of modulators directly into the acidic lumen or membrane of tumor lysosomes *in vivo* requires highly sophisticated drug delivery systems. Moreover, because baseline lysosomal function is indispensable for the survival and homeostasis of normal healthy cells, systemic pharmacological inhibition could induce severe toxicities that potentially mimic the pathology of lysosomal storage disorders. Overcoming these obstacles is crucial for realizing the full therapeutic potential of lysosomal channel modulators in clinical settings.

As our understanding of lysosomal biology deepens, it is increasingly evident that the lysosome is not merely the cellular degradation center but a central hub dictating metabolism, signal transduction, and ultimate cell fate. To overcome current clinical hurdles, future research must shift from simple, systemic channel blockade to more sophisticated functional reprogramming. The key lies in achieving precise, spatiotemporal activation and dynamic regulation of these channels exclusively within the tumor microenvironment. By integrating cross-disciplinary advances in precision medicine, structural biology, and nanotechnology, such as the development of stimuli-responsive nanocarriers, lysosomal ion channel-targeted therapies hold immense promise. Ultimately, they represent a potential breakthrough for next-generation smart therapeutics, offering highly specific and potent strategies to eradicate drug-resistant tumors.
